# Commensal microbe-derived SCFA alleviates atrial fibrillation via GPR43/NLRP3 signaling

**DOI:** 10.7150/ijbs.70644

**Published:** 2022-06-27

**Authors:** Kun Zuo, Chen Fang, Zheng Liu, Yuan Fu, Ye Liu, Lifeng Liu, Yuxing Wang, Xiandong Yin, Xiaoqing Liu, Jing Li, Jiuchang Zhong, Mulei Chen, Li Xu, Xinchun Yang

**Affiliations:** Heart Center & Beijing Key Laboratory of Hypertension, Beijing Chaoyang Hospital, Capital Medical University, Beijing 100020, China.

**Keywords:** Atrial fibrillation, Short-chain fatty acid, NLRP3 inflammasome, Gut microbiota, Atrial remodeling

## Abstract

**Rationale:** Dysbiotic gut microbiota (GM) and NLRP3 inflammasome are proarrhythmic factors in atrial fibrillation (AF). Herein, whether short-chain fatty acid (SCFA) produced from GM fermentation of dietary fiber serving as invisible mediators is yet unclear. Thus, the current study aimed to determine whether SCFA alleviated from NLRP3 signaling-mediated atrial remodeling protects AF development.

**Methods:** First, a cross-sectional study based on the GC-MS metabolomics was performed to explore the association between fecal SCFA levels and AF traits in a cohort consisted of 48 individuals. Then, a well-established mice model fed diet deficient or enriched in dietary fiber was established to elucidate the pathophysiological role of SCFA involved in AF susceptibility, atrial remodeling, and G-protein-coupled receptor 43 (GPR43)/NLRP3 signaling. Finally, the effects of SCFA were verified on HL-1 cells.

**Results:** Fecal SCFA levels were remarkably reduced in AF patients with a declining trend from paroxysmal to persistent AF. Prolonged P wave duration based on surface ECG and increased left atrial diameter gained from echocardiography was identified in low-fiber diet mice but lost in SCFA-supplemented group. Lack of dietary fiber enhanced susceptibility to AF under burst pacing, whereas SCFA might exert a protective effect. The supplementation of SCFA prevented dietary fiber deficiency-upregulated phosphorylation of calmodulin-dependent protein kinase II and ryanodine receptor 2, the disarray fibrosis, collagen expression, and NLRP3 inflammasome activation in atrial tissue. Finally, the AF protective roles of SCFA were identified through GPR43 mediated deactivation of NLRP3 by GPR43 knockdown in HL-1 cells.

**Conclusions:** SCFA derived from dietary fiber fermentation by gut commensals alleviates AF development via GPR43/NLRP3 signaling.

## Introduction

Atrial fibrillation (AF) is one of the most prevalent arrhythmias managed in clinical practice and a significant source of morbidity and mortality 1, 2. Considering the global burden of AF, additional options are required for AF management, where an improved understanding of underlying fundamental mechanisms and novel risk factors is warranted. The key interactome-related nodal point for molecular drivers of AF progression is dealing with systems associated with Ca^2+^/calmodulin-dependent protein kinase-II (CaMKII) and NACHT, LRR, and PYD domain-containing protein 3 (NLRP3), where the intracellular processes related to triggers result from focal ectopic firing and inflammatory signaling 3-6.

In the past decades, increasing evidence has rapidly accumulated, suggesting that gut microbiota (GM) represents a significant environmental factor contributing to the development of several human diseases, accompanied by increased interest in probiotics and prebiotics that modulate the GM 7, 8. Notably, the targeting point is the production of short-chain fatty acid (SCFA) through bacterial fermentation of nondigestible carbohydrates, also known as “dietary fiber,” from the human host diet 9. Deficiency in SCFA production has been associated with several diseases, while increased intake of dietary fibers could alleviate the disease phenotypes in clinical trials 10. In addition to serving as an energy substrate 11, SCFA has potent anti-inflammatory effects on immune system functions 12, 13. The beneficial effects of SCFA have been demonstrated in several disease models, such as hypertension 14, 15, diabetes 16, 17, multiple sclerosis 18, and stroke 19, wherein the SCFA exerts their effects via natural sensors, such as G-protein-coupled receptor (GPR) 43 20, 21.

Our recent studies have described the characteristics of altered structural and functional changes in GM in AF patients based on metagenomic and metabolomic analyses 22-26. We also found that dysbiotic GM in AF is coupled with disrupted SCFA-synthesis-related genes 27. However, the contribution of SCFA to AF, the precise regulatory role of SCFA in AF pathogenesis, and its clinical relevance are yet unclear.

**Figure 1:**
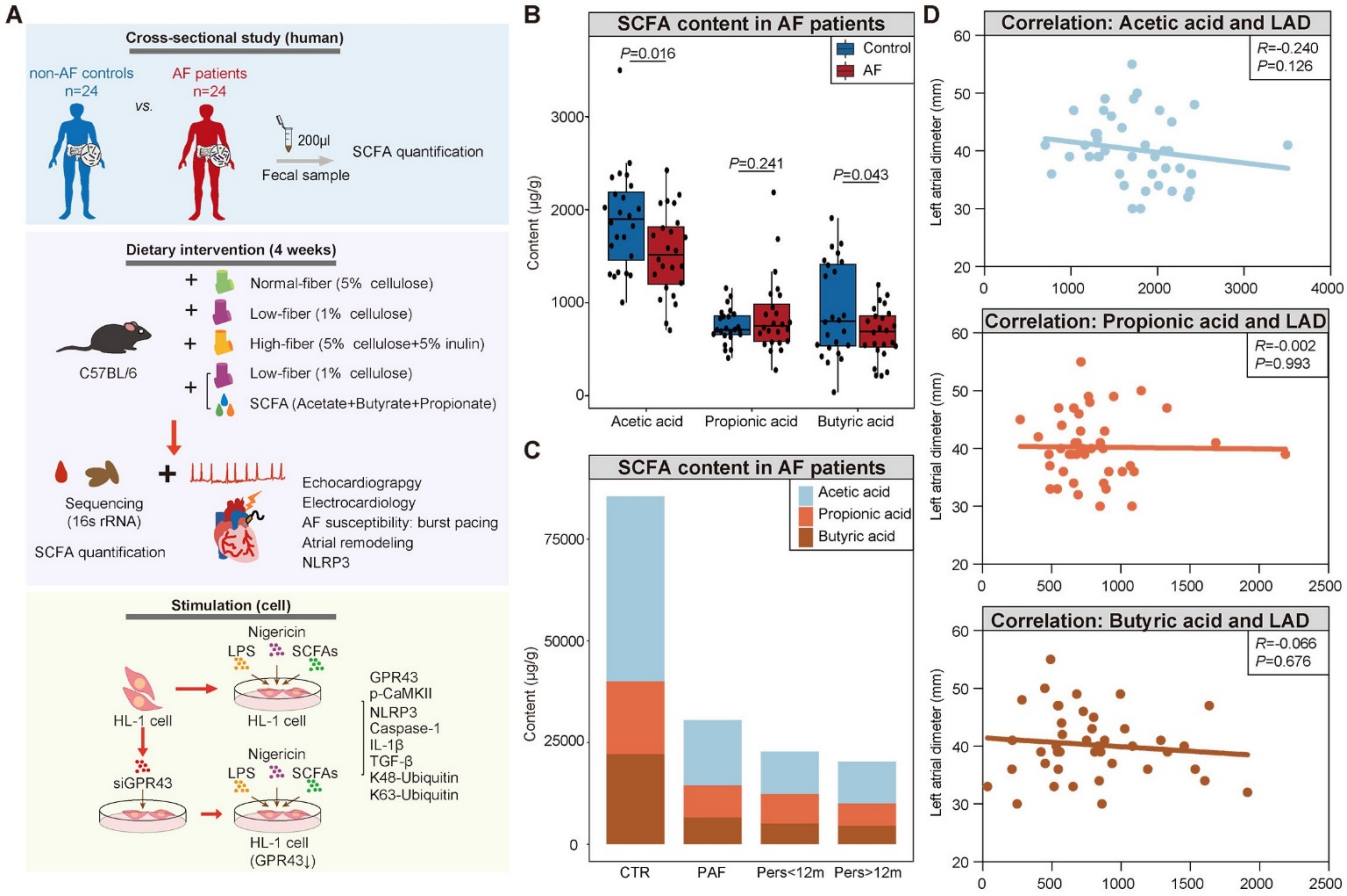
** Decreased fecal SCFA levels in AF patients. (A)** Overview of the study design. **(B)** The box plot showed fecal levels of acetic acid, propionic acid, and butyric acid in non-AF control (CTR) (n=24) and AF patients (n=24). Boxes represent the inter quartile ranges, lines inside the boxes denote medians, and circles are outliers. student's t-test.** (C)** Distribution of SCFA levels according to the progressed types of AF, from non-AF controls (n=24), paroxysmal AF (PAF) (n=9), persistent AF of <12 months (Pers<12m) (n=8) to persistent AF of >12 months (Pers>12m) (n=7). **(D)** Correlation between SCFA levels and left atrial (LA) diameter (LAD). (R=-0.240 *P*=0.126 for Acetic acid; R=-0.002, *P*=0.993 for Propionic acid; R=-0.066, *P*=0.676 for Butyric acid; Spearman correlations).

Based on the prominent role of inflammation 28-30 in AF and recent insights into the anti-inflammatory effects, we hypothesized that the SCFA protects from NLRP3 signaling-mediated atrial remodeling in AF development. In the present study, we aimed to investigate whether gut commensal-derived SCFA alleviates AF pathogenesis, which is mediated by the attenuation of NLRP3 inflammasome activation through GPR43-mediated NLRP3 deactivation. An overview of the study design is illustrated in Figure 1A. First, we performed a cross-sectional study to detect the association between fecal SCFA levels (gas chromatography-mass spectrometry (GC-MS) based-gut metabolomic analysis) and AF traits in a cohort consisting of 24 AF patients and 24 non-AF matched controls. Then, a mouse model fed diet deficient or enriched in dietary fiber leading to GM with decreased or increased capacity for SCFA synthesis was utilized to identify the pathophysiological interaction in the downstream immune and epigenetic effects, including AF susceptibility, electrical substrate, atrial fibrosis, and GPR43 and NLRP3-inflammasome signaling. In addition, the effects of SCFA on the activation of NLRP3 inflammasome through GPR43 were elucidated *in vitro*.

## Material and methods

### Study cohort and sample collection for GC-MS-metabolomics

A total of 48 subjects consisting of 24 healthy individuals and 24 AF patients were enrolled for followed GC-MS analysis. The diagnosis of AF was based on the 2020 European Society of Cardiology guidelines 2. The exclusion criteria were individuals suffering from structural heart disease, coronary heart disease, heart failure, irritable bowel syndrome, acute or chronic infection, autoimmune disease, liver disease, renal disease, and cancer. None of the participants had taken antibiotics or probiotics in the past 1 month. Fresh fecal samples were collected and stored at -80 °C until analysis. All the participants provided informed consent, and the research conformed to the principles outlined in the Declaration of Helsinki.

### Experimental animals and diet intervention

Six- to eight-week-old wild-type male C57BL/6 mice were obtained from the Vital River Laboratory Animal Technology Company (Beijing, China). All mice were kept in a specific pathogen-free environment with the standard temperature (22±2 ℃), humidity (55±5%), and light-dark cycle (12 h/12 h), with free access to rodent chow and water. Female animals were not included to avoid the putative effects of sex steroids. The study protocol was approved by the ethics committee of Capital Medical University. All animal procedures performed conform to the ARRIVE guidelines and the EU Directive 2010/63/EU for animal experiments.

After 7 days of adaptive feeding, mice were randomly divided into four groups: normal-fiber (Control), low-fiber, high-fiber, or low fiber+SCFA. The animals were then maintained on the control diet (5% cellulose), high-fiber diet (5% cellulose+5% inulin), and low-fiber diet (1% cellulose), respectively 31, 32. Mice in the low fiber+SCFA group received sterile water supplemented with sodium propionate (25 mM), sodium butyrate (40 mM), and sodium acetate (67.5 mM) (Sigma-Aldrich) 33, while the other three groups were given only sterile water. All mice were fed for 4 weeks under the above conditions for subsequent experiments. All experiments were blind to group assignment and outcome assessment. All customized fiber-adjusted diets were purchased from Mediscience Ltd. (Yangzhou, China).

### Fecal SCFA quantification based on GC-MS

The fecal samples were homogenized and resuspended in pure water at a ratio of 1:1, followed by centrifugation at 12000 rpm, 4 °C for 15 min. Then, 100 μL of 15% phosphoric acid solution, 20 μL isohexanoic acid solution (375 μg/mL) (internal standard) and 280 μL ethyl ether was added to the supernatant (200 μL), followed by centrifugation at 12000 rpm, 4 °C for 10 min. Then, the supernatant was taken and analyzed by GC-MS (Thermo, USA). The standard curve was used to quantify SCFA (acetic acid, propionic acid, and butyric acid).

### Plasma SCFA quantification based on LC-MS

The plasma samples of mice were mixed with 50% acetonitrile, vortexed for 1 min, sonicated for 30 min at 4 °C and centrifuged at 12000 rpm for 15 min at 4 °C. Then, take 200 μL of supernatant, add 3-NPH (200 mM) and EDC (120 mM; containing 6% pyridine) solution, vortex for 1 min, react at 40 °C for 1 hour, and shake once for 5 minutes. After the completion of the reaction, centrifuge at 12,000 rpm, 4 °C for 15 min to take the supernatant for LC-MS analysis (Waters Acquity UPLC). The standard curve was used to quantify SCFA (acetic acid, propionic acid, and butyric acid).

### *16S* rRNA sequencing and bioinformatic analyses

Fecal specimens of mice were collected through metabolic cages after 4 weeks of intervention and stored at -80 °C. A commercial kit (DP328, Tiangen Biotech, China) was used to extract bacterial DNA. The V4 region of gut microbial *16S* rRNA was characterized by Illumina MiSeq sequencer (250-400 bp). Sequence denoising or operational taxonomic unit (OTU) clustering was performed according to QIIME2 DADA2 analysis process or Vsearch software analysis process. The GreenGene database was utilized for taxonomic annotation. Wilcoxon rank-sum or Kruskal-Wallis test was used to analyze the microbial difference between groups.

### Transthoracic echocardiography

Transthoracic echocardiography using a high-frequency ultrasound imaging system at 30 MHz (Vevo 2100 ultrasound instrument, FUJIFILM VisualSonics, Canada) was performed to evaluate cardiac function and chamber dimensions after anesthesia with 1.5%-2% isoflurane. Left atrial (LA) diameter, left ventricular chamber dimension (LVID) at end-diastole and ejection fraction (EF) were derived by two-dimensional M-mode. Images were recorded digitally for further analysis. At least three cardiac cycles were measured and averaged.

### AF inducibility by transesophageal burst pacing

Mice underwent anesthesia with 1.5%-2% isoflurane and electrodes were fixed on limbs to yield a surface II-lead echocardiogram (ECG). AF was induced via transesophageal burst rapid pacing with a 1.1-Fr octapolar catheter (EPR-800, Millar Instruments, USA) 34. Each mouse was stimulated five times continuously. AF was defined as an abnormal ECG with an irregular atrial rhythm, P wave loss, and irregular R-R intervals, persisting for at least 1 s. Successful AF induction was defined as at least 2/5 episodes of AF. The percentage of mice with induced AF was displayed as AF inducibility, which was verified by an experienced cardiac physiologist.

### Histological examinations

Atrial tissue samples from mice were fixed in 4% paraformaldehyde, embedded in paraffin, and sectioned into 5-μm slices. Then, the sections were stained with Masson's trichrome, and Sirius Red to detect atrial fibrosis, and collagen, respectively. The images were observed and captured by a Pixera Pro600EX camera on a VANOX-S microscope (Olympus Co., Tokyo, Japan) and were analyzed with the ImageJ software (Version 1.8.0).

### Immunohistochemistry and immunofluorescence staining

Mice atrial sections were incubated with primary antibody against α-SMA (Proteintech, USA) at 4 °C overnight, followed by HRP conjugated secondary antibody. The immunoreaction was visualized using diaminobenzidine (DAB), and images were obtained by a Pixera Pro600EX camera on a VANOX-S microscope. For immunofluorescence, the atrial tissue sections of mice were stained with primary antibodies against connexin 43 (rabbit; Proteintech, USA) and N-cadherin (mouse; Proteintech) at 4 °C overnight. After incubation with secondary antibodies conjugated with FITC (fluorescein isothiocyanate) (Alexa Fluor® 488 and Alexa Fluor® 594, respectively) and DAPI (4',6-diamidino-2-phenylindole) counterstaining, images were captured using an Olympus IX51 microscope (Olympus).

### Cell culture

Murine atrial-derived HL-1 cardiomyocytes were purchased from the BNCC (BNCC288890, China) and cultured in Dulbecco's modified eagle medium (DMEM) containing 10% fetal bovine serum (FBS) in a humidified incubator at 37 °C under 5% CO_2_. After pre-treatment with SCFA including sodium propionate (0.5 mM), sodium butyrate (0.5 mM), and sodium acetate (10 mM) (Sigma-Aldrich) for 2 h, the cells were stimulated with LPS (500 ng/mL) for 4 h and then nigericin (10 μM) for 1 h to activate the NLRP3 inflammasome 35.

### Knockdown of GPR43

HL-1 cells were transfected with GPR43 siRNA or control siRNA (Santa Cruz Biotechnology, USA) using Lipofectamine^®^ 3000 (Invitrogen, USA), according to the manufacturer's instructions. After culturing for 48 h, the cells were stimulated according to their groups for subsequent experiments.

### Western blot analysis

Whole protein extract from mice atrial tissue and the cultured HL-1 cells was estimated by BCA assay. An equivalent of 40 μg protein was separated by SDS-polyacrylamide gel electrophoresis (SDS-PAGE) and transferred to nitrocellulose membranes by electroblotting. Subsequently, the membranes were blocked with 5% skimmed milk for 1 h and probed with primary antibodies against phospho-CaMKII, phospho-RYR2, CaMKII, RYR2, TGF-β, α-SMA, collagen I, GPR43, IL-1β, NLRP3, Ubiquitin (linkage-specific K48), ubiquitin (linkage-specific K63), β-tubulin, and GAPDH at 4 °C overnight, followed by secondary fluorescence antibodies at ambient temperature for 1 h. The immunoreactive bands were visualized using ImageQuant LAS 4000 (GE Healthcare, Baie d'Urfe). GAPDH and β-tubulin were used as an endogenous control. Antibodies were obtained from Cell Signaling Technology (Beverly, MA, USA), Santa Cruz Biotechnology (Santa Cruz, CA, USA), ABclonal Technology Co.,Ltd. (Wuhan, China) and Abcam Inc. (Cambridge, MA, USA), respectively.

### Statistical analysis

Continuous variables were expressed as mean±standard deviation (SD) (population-based data), mean±standard error of the mean (SEM) (animal-based data), or median (quartile). Student's *t*-test or Mann-Whitney *U* test was used to measure the difference in normally or non-normally distributed data, respectively. The Shapiro-Wilk test was performed to examine the normality. The categorical variables were shown as the number or a percentage and were compared using the chi-square test. All statistical analyses were carried out using SPSS 25.0 (SPSS, USA) or R software (version 2.15.3). Two-sided *P*<0.05 was considered statistically significant. All experiments were repeated at least three times.

## Results

### Decreased fecal SCFA levels in AF patients

Although the disrupted SCFA synthesis-related genes, characterized by decreased enzymatic genes and harboring species, have been identified in AF patients based on previous metagenomic analyses 27, the actual levels of SCFA have not yet been obtained in AF patients. Therefore, the fecal levels of SCFA were measured through GC-MS quantification in a cohort comprising 24 AF patients and 24 matched healthy individuals (CTR) (Table S1). And there was no significant difference in baseline characteristics between the two groups. The results showed a decline in SCFA level in fecal samples from AF patients (3559.68±986.59 μg/g v*s*. 3060.67±655.34 μg/g for CTR *vs*. AF, *P*=0.045) (Figure 1B), including acetic acid (1891.71±544.16 μg/g v*s*. 1530.81±456.46 μg/g for CTR *vs*. AF, *P*=0.016), butyric acid (920.71±511.53 μg/g *vs*. 671.60±277.92 μg/g for CTR *vs*. AF, *P*=0.043), and propionic acid (747.25±184.48 μg/g *vs*. 858.26±416.34 μg/g for CTR *vs*. AF, *P*=0.241). Interestingly, a declining trend of SCFA levels was observed among the progressed types of AF, from non-AF controls, paroxysmal AF (PAF), persistent AF (psAF) of <12 months (Pers<12m) and psAF of >12 months (Pers>12m) (Figure 1C).

Considering the potential interaction between deficiency of SCFA and AF development, the correlation between SCFA levels and left atrial (LA) anteroposterior diameter, an indicator of atrial fibrosis responsible for AF perpetuation, was further described. LA anteroposterior diameter (LAD) was negatively correlated with acetate level without statistical significance (Figure 1D), possibly due to the limitation of sample size. These findings indicated that deficiency of SCFA level is closely associated with AF development.

### Dietary fiber alters GM structure and SCFA production

**Figure 2:**
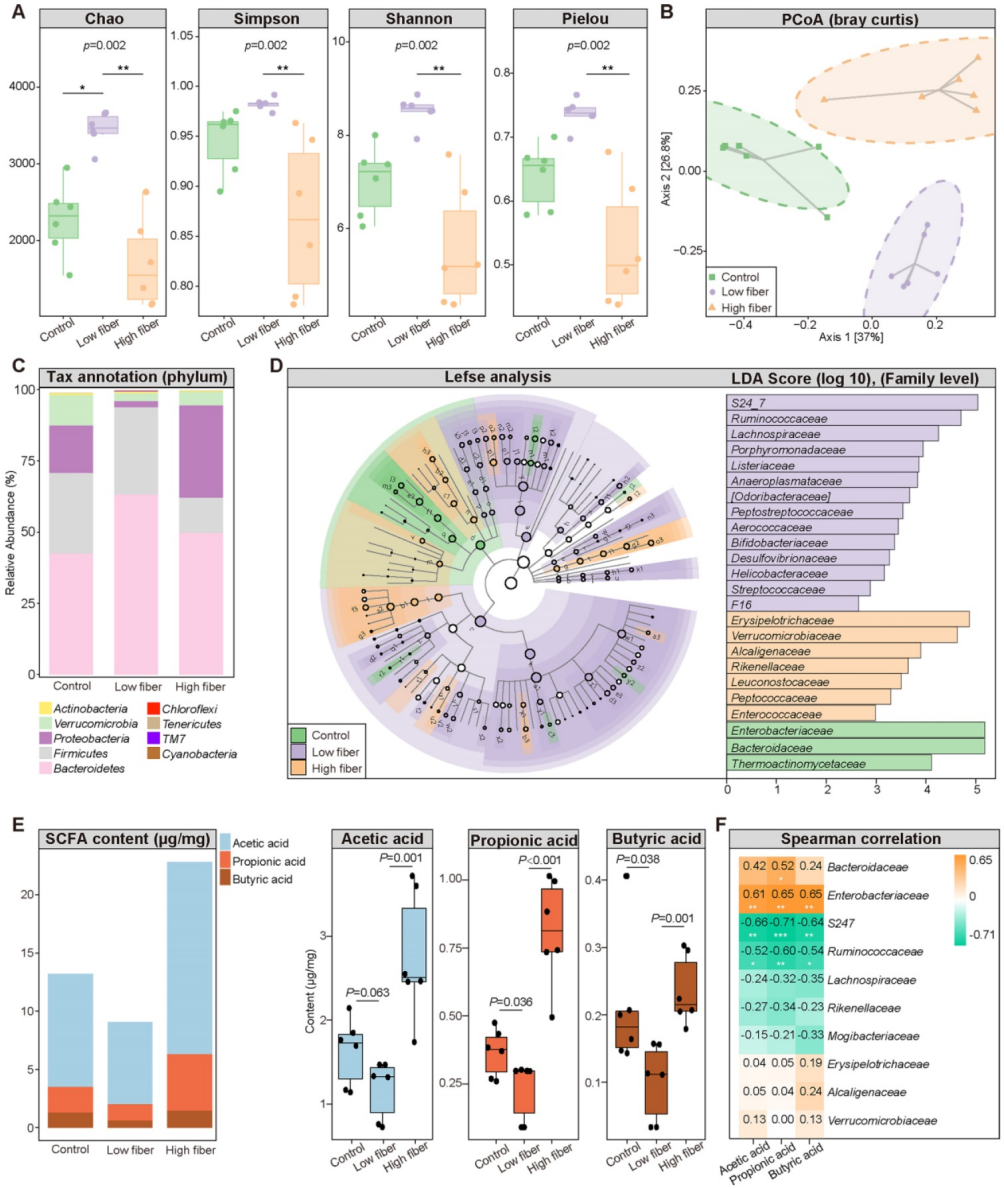
** Dietary fiber alters GM structure and SCFA production. (A)** The α-diversity including Chao, Simpson, Shannon and Pielou index in the normal-fiber control (*green*), low-fiber (*purple*), and high-fiber (*orange*) group. Boxes represent the inter quartile ranges, the inside line or points represent the median, and circles are outliers. n=6 for each group, Kruskal-Wallis test. **(B)** Principal coordinates analysis (PCoA) based on bray curtis distance. The green squares represent normal-fiber control, orange triangles refer to high-fiber, and purple circles denote low-fiber. n=6 for each group. **(C)** Phylum-level taxonomic abundance and proportion for the normal-fiber control (*right*), low-fiber (*middle*), and high-fiber (*left*) group, where different taxa are differentiated by color. n=6 for each group. **(D)** Cladogram showing different taxonomic compositions among the normal-fiber control (*green*), low-fiber(*purple*), and high-fiber group (*orange*) based on the linear discriminant analysis (LDA) effect size (LefSe) analysis. Histogram of LDA scores showing differentially abundant taxon (family level). The taxon with |LDA score (log10)| >2 and *P* < 0.05 are listed, n=6 for each group. **(E)** Distribution of fecal SCFA levels in control (n=6), low-fiber (n=6), and high-fiber groups (n=6). student's t-test. **(F)** Spearman's correlation analysis between fecal SCFA levels and the top-10 most abundant genera in high-fiber group. *Green*, negative correlation; *yellow*, positive correlation; n=6 for each group; *, *P* < 0.05; **, *P* < 0.01, ***, *P* < 0.001.

To determine the influence of the SCFA-derived from gut commensals on AF, we fed mice normal (5% cellulose), low (1% cellulose), or high (5% cellulose+5% inulin) fiber diet, respectively, for 4 weeks. The 16S rRNA sequencing results showed that the GM diversity (Figure 2A), including Chao richness, Simpson index, Shannon index, and Pielou evenness, was significantly increased in the low-fiber group than in the other two groups, accompanied by altered global taxonomic composition (Figure 2B-D). Meanwhile, SCFA supplement could alter the GM structure in mice fed with low fiber diet (Figure S2).

Moreover, decreased fecal SCFA levels were quantified in the low-fiber group (Figure 2E) (acetic acid: 1.63±0.40 μg/mg *vs*. 1.18±0.35 μg/mg *vs*. 2.76±0.76 μg/mg for Control *vs*. low-fiber *vs*. high-fiber group, *P*=0.063, *P*=0.001; butyric acid: 0.21±0.10 μg/mg *vs*. 0.10±0.06 μg/mg *vs*. 0.24±0.05 μg/mg for Control *vs*. low-fiber *vs*. high-fiber group, *P*=0.038, *P*=0.001; propionic acid: 0.37±0.09 μg/mg *vs*. 0.23±0.11 μg/mg *vs*. 0.81±0.20 μg/mg for Control *vs*. low-fiber *vs*. high-fiber group, *P*=0.036, *P<*0.001), and multi-omics analyses revealed a disrupted balance between recognized SCFA-producing probiotics such as *Bacteroides* and the enriched pro-inflammatory *Ruminococcus* in the low-fiber group (Figure S1) 24, 36. *Akkermansia*, a beneficial genus reported to metabolically support the growth of SCFA-producers, was enriched in the high-fiber group (Figure S1) 37. Moreover, the same tendency of SCFA level in plasma was detected (Figure S3). These results showed the direct effect of dietary fiber on gut flora and SCFA production.

### Lack of dietary fiber derived-SCFA alters the physiological index of the left atrium

**Figure 3:**
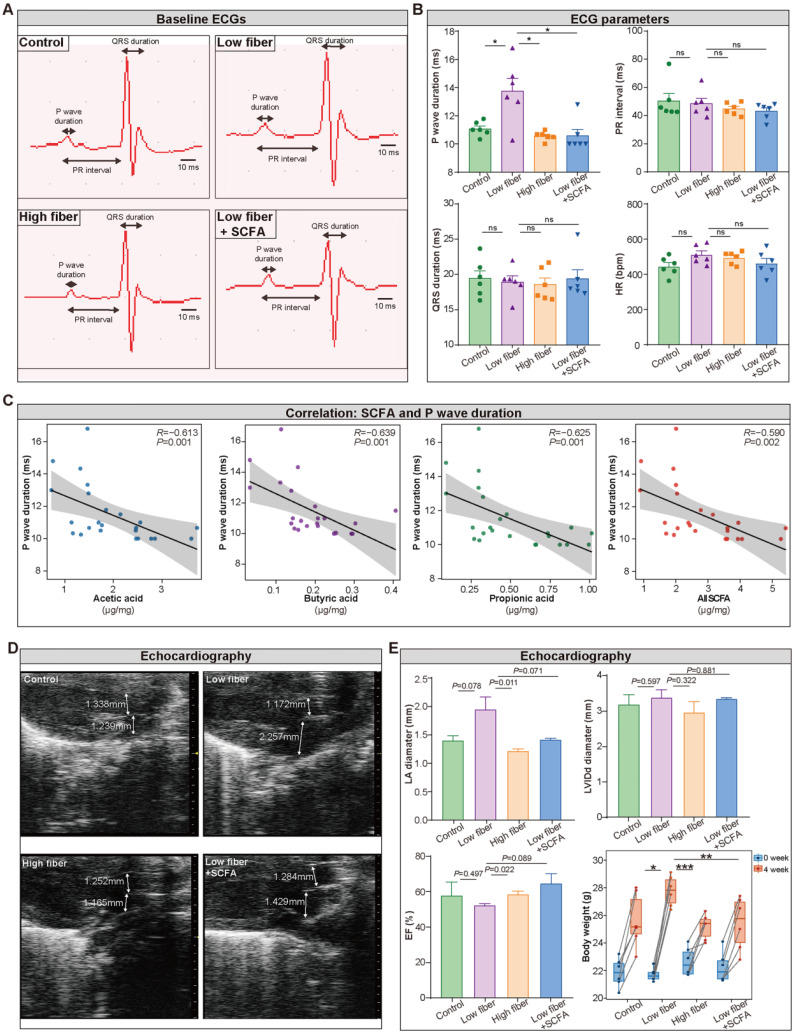
** Lack of dietary fiber derived-SCFA alters the physiological index of the left atrium. (A)** Representative images of baseline surface ECG in the normal-fiber control, low-fiber, high-fiber, and low fiber+SCFA group. P wave duration, PR interval and QRS duration is indicated by arrows. **(B)** P wave duration, PR interval, QRS duration, Heart rate (HR) in control (n=6), low-fiber (n=6), high-fiber (n=6) and low fiber+SCFA (n=6) groups. **(C)** Correlation between SCFA levels and P wave duration. (R=-0.613, *P*=0.001 for acetic acid; R=-0.639, *P*=0.001 for butyric acid; R=-0.625, *P*=0.001 for propionic acid; Spearman correlations). n=6 for each group. **(D)** Representative images of mice left atrium (LA) and aorta (AO) diameter measured by transthoracic echocardiography.** (E)** Bar plots of LA diameter, LVIDd diameter, ejection fraction (EF) (n=3 for each group) and alterations of body weight (n=6 for each group) between 0 week and 4 week in control, low-fiber, high-fiber and low fiber+SCFA group. *, *P*<0.05; **, *P*<0.01; ***, *P*<0.001; ns, no significance, data are mean±SEM, student's t-test.

To detect the influence of SCFA derived from dietary fiber on atrial electrical conduction and cardiac structure and function, surface electrocardiography (ECG) and transthoracic echocardiography were performed among the four groups. The waveform of lead II was extracted (Figure 3A), and the heart rate, PR interval, and QRS duration, reflecting the conduction function of the atrial-ventricular node and left ventricle, were similar in the four groups (Figure 3B). Notably, prolonged P wave duration (Figure 3B), a potent indicator for disorganized and chaotic atrial electrical conduction, was detected in mice fed a low-fiber diet (11.07 *vs*. 13.75 *vs*. 10.56 ms for CTR *vs*. low-fiber *vs*. high-fiber group; *P*=0.015, *P*=0.015). This phenomenon could be reversed by supplementary SCFA (13.75 *vs*. 10.58 ms for low-fiber *vs*. low fiber+SCFA group, *P*=0.010). In addition, P wave duration was negatively correlated with SCFA levels (Figure 3C), including acetic acid (R=-0.613, *P*=0.001), butyric acid (R=-0.639, *P*=0.001), and propionic acid (R=-0.625, *P*=0.001).

Meanwhile, echocardiography (Figure 3D) showed increased left atrial diameter (low-fiber *vs*. high-fiber group; *P*=0.011; Figure 3E) and impaired ejection fraction (low-fiber *vs*. high-fiber group; *P*=0.022; Figure 3E) in low-fiber diet mice but not in high-fiber diet or SCFA-supplemented mice. These findings provided preliminary evidence that dietary fiber derived-SCFA might be involved in atrial remodeling.

### Lack of dietary fiber-derived SCFA increases AF susceptibility

**Figure 4:**
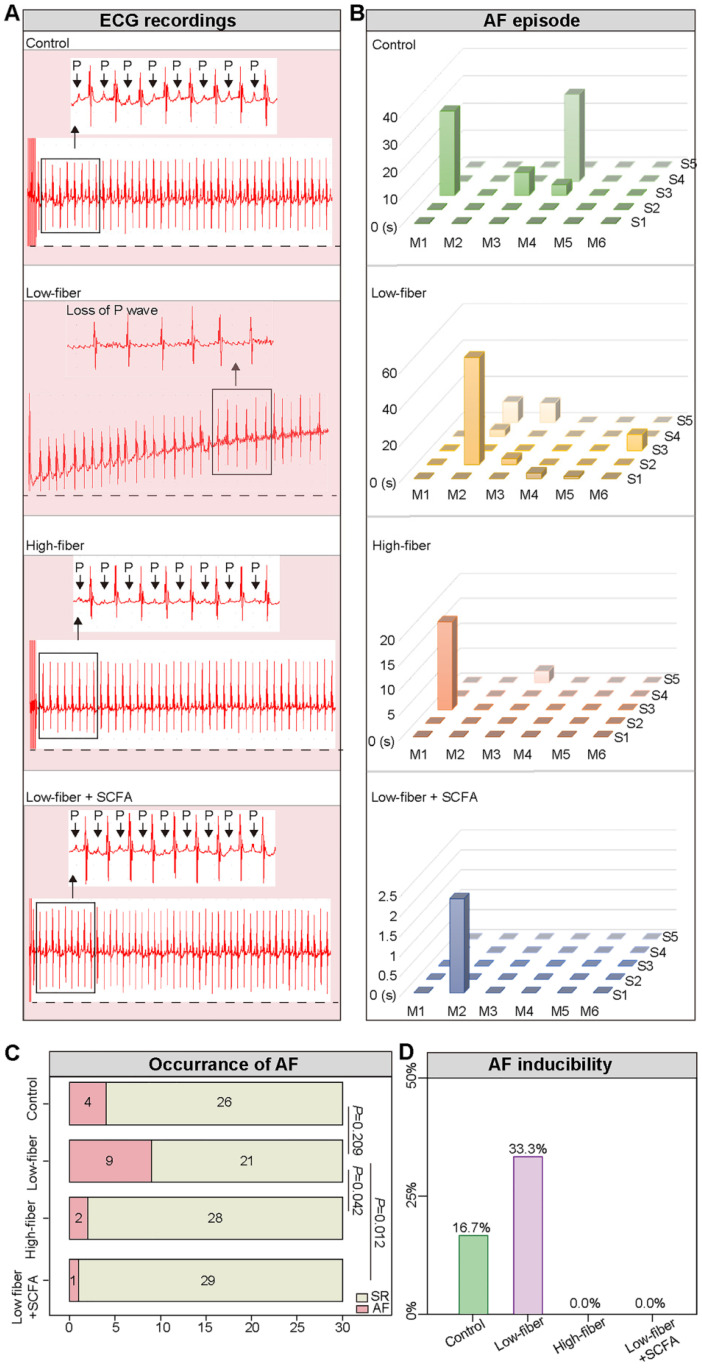
** Lack of dietary fiber-derived SCFA increases AF susceptibility. (A)** Representative image of surface ECG recordings during burst pacing in control, low-fiber, high-fiber and low fiber+SCFA group. **(B-D)** AF inducibility and AF duration in control (n=6), low-fiber (n=6), high-fiber (n=6) and low fiber+SCFA group (n=6). Each mouse was stimulated five times continuously. Chi-square test for C.

To evaluate AF inducibility and duration in mice fed different types of dietary fiber, transesophageal burst pacing was performed. Consequently, no episode of atrial arrhythmia was observed in either of the groups at baseline, and representative ECGs following burst pacing are depicted in Figure 4A, B. Burst pacing induced an AF rate of 9/30 (30.00%) in low-fiber, 2/30 (6.67%) in high-fiber, and 1/30 (3.33%) in SCFA-supplementary mice (Figure 4C). The successful induction of AF in mice was defined as the occurrence of AF at least 2/5 times. Mice in the low-fiber group (2/6; 33.33%) had a higher AF inducibility compared to the control (1/6; 16.67%), high-fiber (0/6), or SCFA-supplementary group (0/6) (Figure 4D). Together, the above data suggested that lack of dietary fiber increased AF susceptibility, whereas SCFA exerted a partially protective effect.

### Lack of dietary fiber derived-SCFA exacerbates the disturbed conduction in the atrium

**Figure 5:**
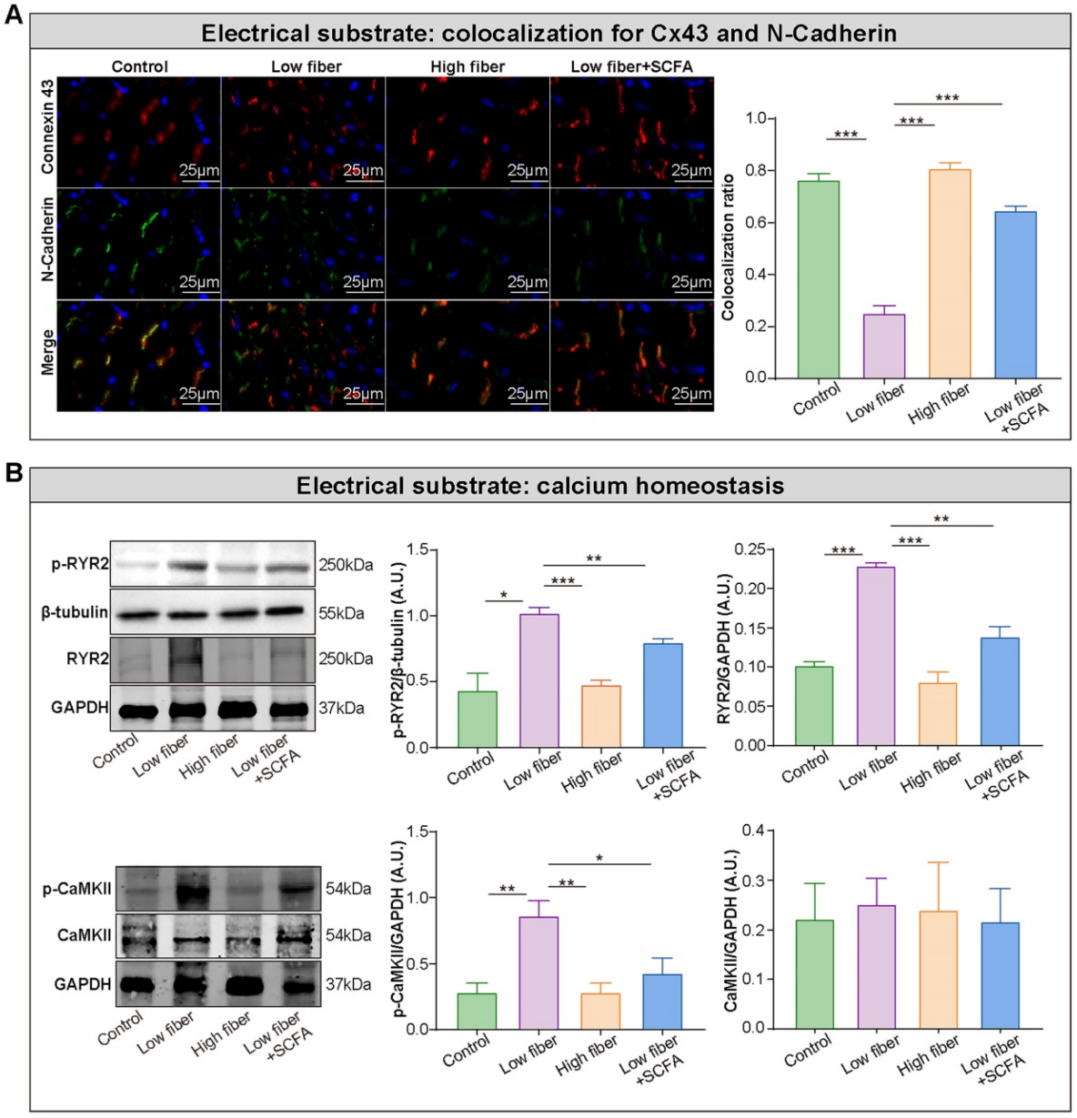
** Lack of dietary fiber derived-SCFA exacerbates the disturbed conduction in the atrium. (A**) Left panel, representative immunofluorescent staining for connexin 43 (*red*) and N-cadherin (*green*) in the atrium of low-fiber, compared with control, high-fiber or low fiber+SCFA group. Right panel, co-localization ratios based on Pearson's correlation coefficients for connexin 43 and N-cadherin signals. ***, *P*<0.001; n=3 for each group; data are mean±SEM. **(B)** Representative western blot images (left panel) and related analyses (right panel) of atrial p-CaMKII, CaMKII, RyR2, and p-RyR2, with GAPDH or β-tubulin as an endogenous control. n=4-5; *, *P*<0.05; **, *P*<0.01; ***, *P*<0.001; data are mean±SEM; A.U., arbitrary units; student's t-test.

Electrical remodeling promotes a reentry-prone milieu, the electrophysiological substrate for AF, which is caused by dysfunction of ion channels, disrupted gap junction distribution, and lost colocation for the connexin hemi-channels connexin 43 (Cx43) and N-cadherin 38-40. In the normal- and high-fiber diet groups, Cx43 and N-cadherin gap junctions were colocated at cell termini, with minimal side-to-side interconnections (Figure 5A and Figure S4). Conversely, the distribution patterns of Cx43 and N-cadherin were markedly altered in the low-fiber group with intense lateral deposition by non-colocation, (Figure 5A and Figure S4). Notably, the non-colocation was alleviated in the SCFA-supplementary group (Figure 5A and Figure S4). Furthermore, the immunoblotting results suggested that the supplementation of SCFA prevents dietary fiber deficiency-mediated Ca^2+^ handling disruption characterized by upregulated expression levels of phosphorylated CaMKII and CaMKII-related phosphorylation of ryanodine receptor 2 (RyR2) in the atrium (Figure 5B). These findings preliminary suggested the protective effects of dietary fiber-derived SCFA on atrial Cx43/N-cadherin location and CaMKII-mediated phosphorylation of RyR2, which might relate to the stability of atrial electrical substrate.

**Figure 6:**
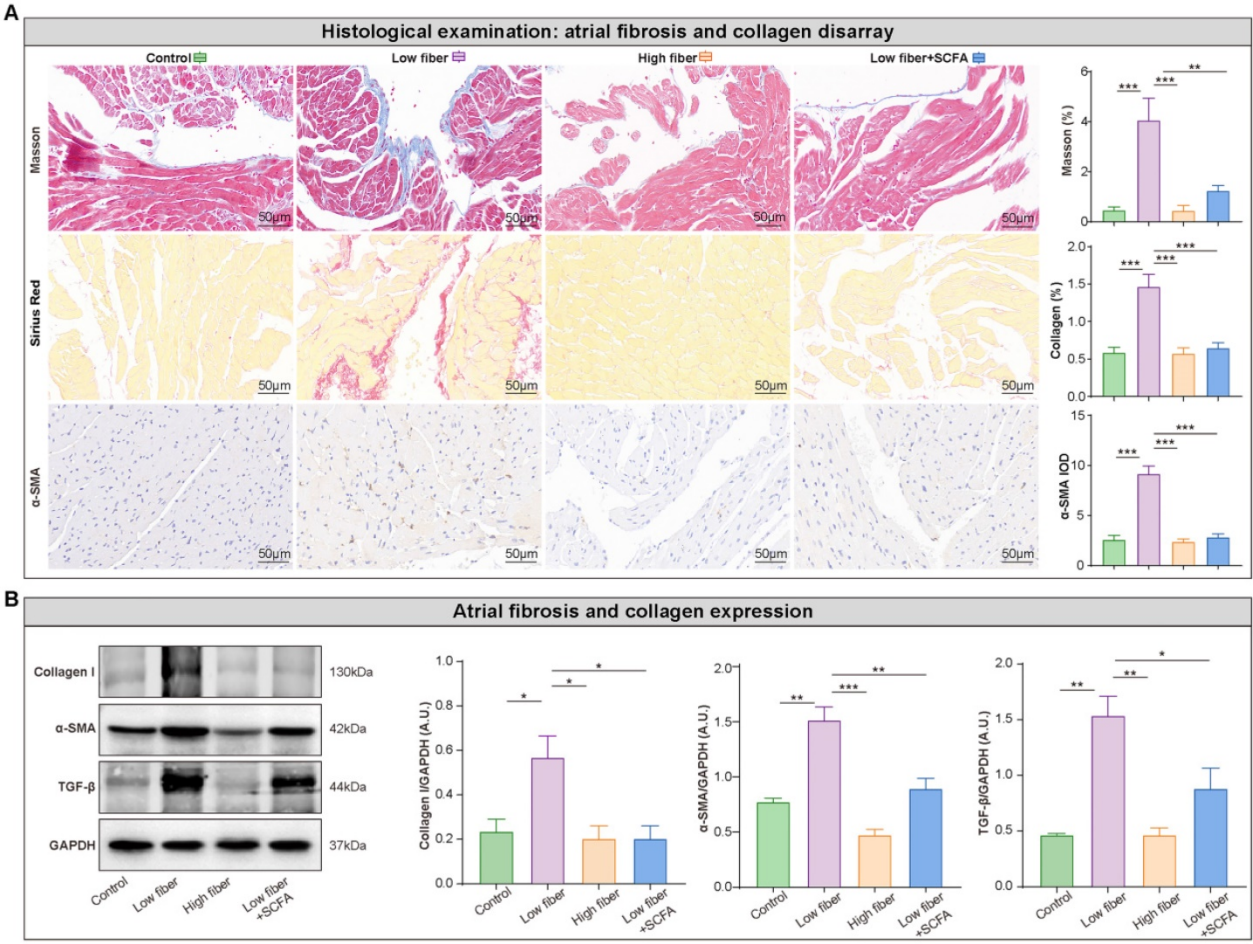
** Lack of dietary fiber derived-SCFA aggravates atrial fibrosis. (A)** Representative Masson trichrome, Sirius Red, and immunohistochemical staining for fibrosis disarray (*blue*), collagen deposition (*red*) and α-SMA expression (*brown*) in the atrium. **, *P*<0.01; ***, *P*<0.001; n=3 for each group; data are mean±SEM; student's t-test. **(B)** Representative western blot of collagen I, α-SMA and TGF-β, with GAPDH as an endogenous control. A.U., arbitrary units; n=4-5; *, *P*<0.05; **,* P*<0.01; ***, *P*<0.001; data are mean±SEM; student's t-test.

Atrial structural remodeling, primarily including tissue fibrosis, is associated with conduction abnormalities that create a substrate for AF maintenance 38, 39. Disarray fibrosis and collagen expression showed a remarkable progression in the atrial tissue in low-fiber diet mice, based on Masson trichrome and Sirius red staining, which could be alleviated by supplementary SCFA (Figure 6A). This phenomenon was consistent with the protein levels of several fibrosis-related markers, such as collagen I, TGF-β, and α-SMA (Figure 6B). Taken together, these findings partially explained the pathophysiological process between dietary fiber-derived SCFA, heterogenic atrial conduction, and AF susceptibility.

### SCFA alleviates NLRP3 activation observed in mice fed low-fiber diet

**Figure 7:**
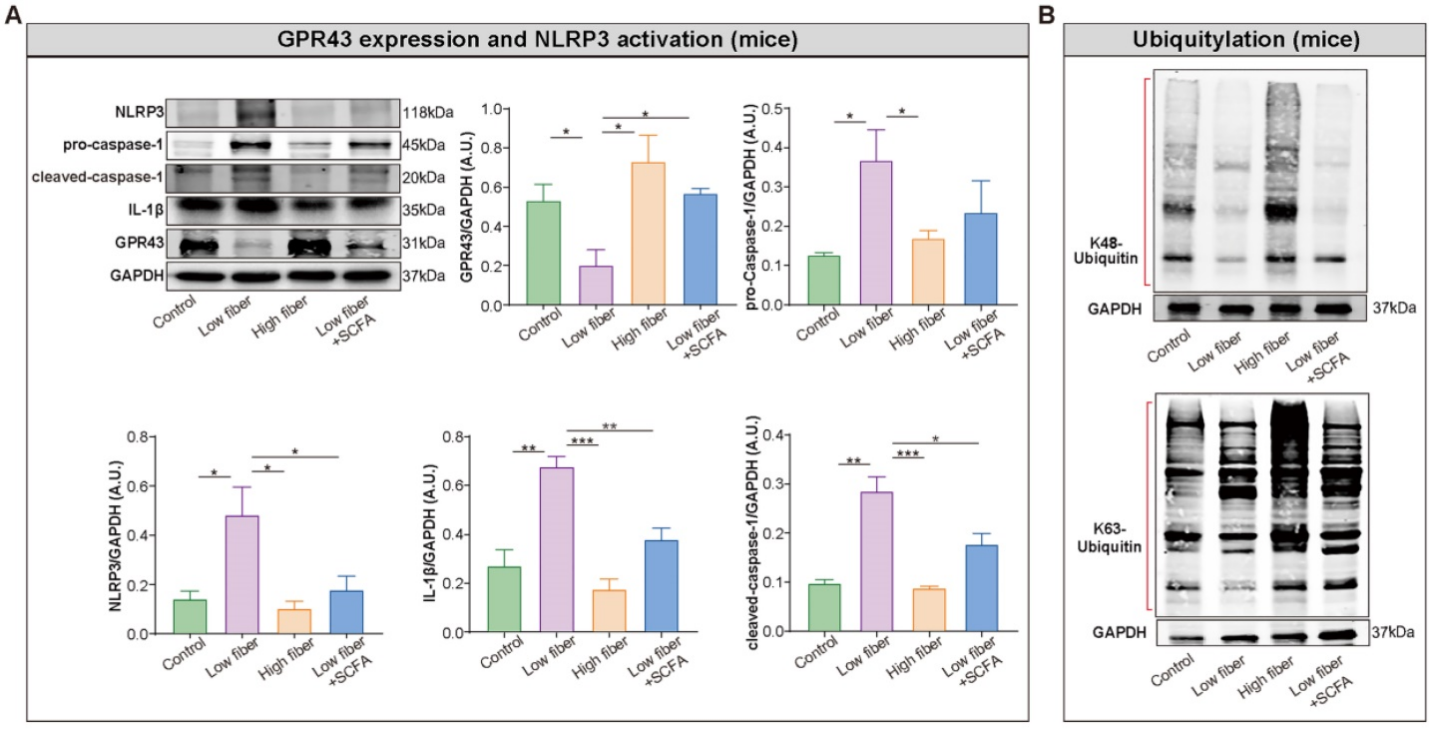
** Enrichment of dietary fiber derived-SCFA attenuates NLRP3 activation through GPR43 signaling.** Representative western blot of GPR43, NLRP3, pro-caspase-1, cleaved-caspase-1 and IL-1β **(A)**, as well as K48- and K63-linked ubiquitylation **(B)** in atrial tissue of mice fed with normal, low-fiber, high-fiber and low fiber+SCFA, with GAPDH as an endogenous control. A.U., arbitrary units. *, *P*<0.05; **, *P*<0.01; ***, *P*<0.001; n=3-6; data are mean±SEM; student's t-test.

Recent studies provided an in-depth insight into the anti-inflammatory properties of SCFA via its sensor GPR43 in different tissues and cell types. Thus, we hypothesized that SCFA exerts a protective effect on NLRP3 inflammasome activation. Firstly, the high level of NLRP3, pro-caspase-1, cleaved caspase-1 and interleukin-1 beta (IL-1β) was identified in the atrium of low-fiber group (Figure 7A), indicating the correlation between low-fiber diet and NLRP3 activation, although the potential mechanism is not yet elucidated. Next, the increased K48- and K63-linked ubiquitylation, an indicator for autophagic degradation and deactivation, was identified in atrial tissue of mice fed high-fiber diet or SCFA-supplemented group, suggesting a possible alleviation effect of SCFA in NLRP3 activation (Figure 7B). In addition, the upregulated protein level of GPR43, a natural sensor of SCFA, was observed in high-fiber and SCFA-supplemented groups (Figure 7A), indicating a regulatory role of GPR43 that requires further investigation. Taken together, these preliminary results described the probable effect that dietary fiber-derived SCFA prevents NLRP3 inflammasome activation via GPR43 by autophagic degradation through K48- and K63-linked ubiquitylation, and thus exerts a protective role on AF.

### SCFA attenuates inflammasome activation and related atrial remodeling through GPR43-signaling in HL-1 cells

**Figure 8:**
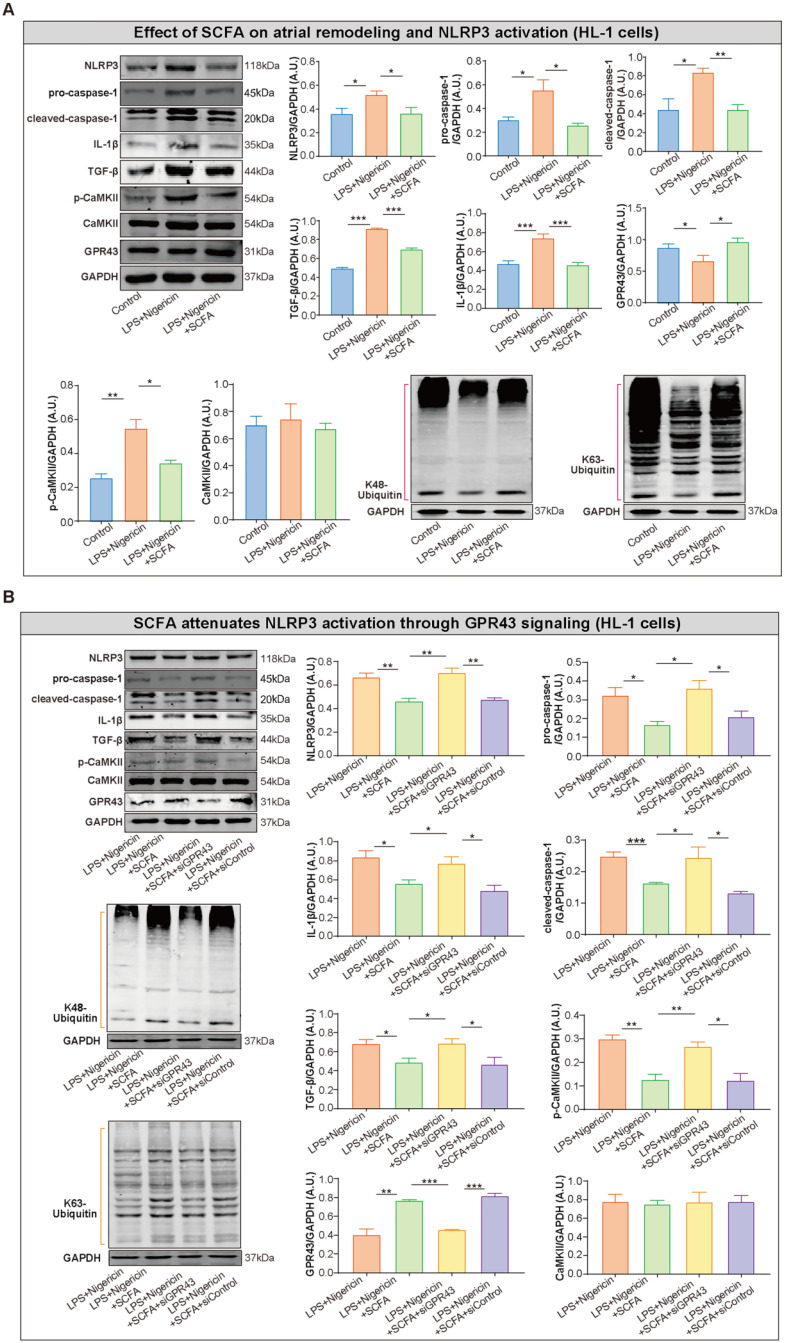
** SCFA attenuates NLRP3 activation through GPR43 signaling in HL-1 cells. (A)** Representative western blot of GPR43, p-CaMKII, CaMKII, NLRP3, pro-caspase-1, cleaved-caspase-1, IL-1β, K48- and K63-linked ubiquitylation and TGF-β in HL-1 cells treated with or without LPS (500 ng/ml), nigericin (10 µM) and SCFA, including sodium propionate (0.5 mM), sodium butyrate (0.5 mM) and sodium acetate (10 mM) (n=3-9). **(B)** Representative western blot of GPR43, p-CaMKII, CaMKII, NLRP3, pro-caspase-1, cleaved-caspase-1, IL-1β, ubiquitination with both K48- and K63-linked ubiquitin chains and TGF-β in HL-1 cells treated with or without siGPR43, siControl, LPS, nigericin and SCFA (sodium propionate, sodium butyrate and sodium acetate) (n=3-5). GAPDH as an endogenous control. A.U., arbitrary units. *, *P*<0.05; **, *P*<0.01; ***, *P*<0.001; data are mean±SEM; student's t-test.

To further explore the inhibitory effect of SCFA on NLRP3 activation, lipopolysaccharide (LPS)-primed HL-1 cells were treated with SCFA before nigericin stimulation. Subsequently, the protein levels of NLRP3, pro-caspase-1, cleaved caspase-1 and IL-1β in the HL-1 cells were estimated. We found that SCFA suppressed the LPS/nigericin-induced IL-1β production and caspase-1 activation (Figure 8A). Next, the mechanism by which SCFA mediates NLRP3 suppression is analyzed. Herein, we found that SCFA promoted GPR43 expression and ubiquitination with both K48- and K63-linked ubiquitin chains in HL-1 cells. Moreover, alleviated CaMKII phosphorylation was observed when the inflammasome activation was attenuated through GPR43-mediated NLRP3 deactivation in HL-1 cells (Figure 8A). However, these effects of SCFA on the inflammasome were diminished in cells treated with siGPR43 before LPS and nigericin stimulation (Figure 8B). Of note, pre-treatment with KN93 (10 μmol/L), a selective inhibitor of CaMKII, could abolish LPS/nigericin-induced NLRP3 activation (Figure S5). Collectively, these data indicated that SCFA regulates the activation of NLRP3 inflammasome via GPR43 and CaMKII, which preliminarily revealed the potential mechanism of SCFA-mediated NLRP3 inflammasome attenuation.

## Discussion

This study provides novel pathophysiological insights into the association between dietary fiber, SCFA, NLRP3 signaling and AF, which would guide novel treatment options to delaying AF progression. We demonstrated that lack of dietary fiber-derived SCFA contributes to an increased AF susceptibility, disrupted atrial electrical substrate, and atrial fibrosis, which has not yet been reported. Moreover, we provided initial support revealing that SCFA attenuates the NLRP3 inflammasome activation via GPR43 signaling, suggesting it to be the mechanism underlying SCFA-mediated NLRP3 inflammasome attenuation.

The gut commensals, serving as a virtual endocrine system, communicate with distal organs through metabolism-dependent pathways. SCFAs are a major class of bacterial metabolites mainly produced by the bacterial fermentation of otherwise indigestible fibers 41. In addition to serving as energy substrates, SCFA has potent anti-inflammatory effects on various systems 11, 13. A decrease in bacterial SCFA is associated with several diseases. For example, SCFA-producing bacteria alleviate post-stroke neurological deficits and inflammation 19. Interestingly, propionate administration influences T helper cell homeostasis, thereby reducing cardiac hypertrophy and fibrosis, susceptibility to ventricular arrhythmias, and atherosclerotic lesion burden 15. A recent study based on metagenomic data analysis of AF cohort also revealed that decreased SCFA-synthesized enzymatic genes and harboring species are coupled with AF 27. We also provided direct evidence of decreased fecal SCFA levels in AF patients based on targeted metabolomic measurement. Consistently, mice with low SCFA levels exhibited increased vulnerability to AF, which could be alleviated by SCFA supplement.

The current results showed that GPR43 expression might be regulated in an SCFA-dependent manner. SCFA has been found to promote the mRNA expression of GPR43 in the colon of mice 42. In human studies, decreased plasma SCFA was accompanied by lower GPR43 expression in peripheral white blood cells 43. Decreased GPR43 expression was disclosed in psoriatic skin, which could be restored by the application of butyrate 44. And further studies are needed to determine the specific roles of dietary fiber-derived SCFA on GPR43 expression.

Increasing evidence of NLRP3-inflammasome signaling as a central proarrhythmic mediator of multiple pathophysiological signals in AF 45. Mice with cardiomyocyte-restricted constitutive activation of the NLRP3-inflammasome show increased atrial ectopy associated with Ca^2+^-handling abnormalities due to upregulated RyR2 expression, reentry-promoting action potential duration abbreviation (because of increased atrial-selective ultra-rapid delayed-rectifier K^+^-current and acetylcholine-activated inward-rectifier K^+^-current), and atrial fibrosis 3. A recent study described the role of disordered GM-atrial NLRP3 inflammasome axis in the pathogenesis of age-related AF, that the aging accompanied by elevated LPS and hyperglycemia was speculated as the potential factor which promotes the aged microbiota-induced AF. And this report provides evidence about the promoting effect of dysbiotic gut bacteria and atrial NLRP3 inflammasome in the pathogenesis of AF 46.

The current results showed that the activated GPR43 was followed by the suppressed p-CaMKII and NLRP3 signaling, although the potential mechanism remains unknown. It has been well-established that activation of CaMKII played a pivotal role in the pathogenesis of severe heart conditions including the activation of NLRP3 in cardiomyocytes 47, 48. And our data indicated that CaMKII activation might represent a nodal point for NLRP3 activation. Notably, the above results did not identify the specific subunit or related molecules for GPR43 and downstream of CaMKII, thus future study is necessary to understand the influence of GPR43/CaMKII on critical factors in NLRP3 signaling.

In the current study, the relationship between a low dietary fiber diet derived-SCFA and NLRP3 activation has been confirmed, but the bridge of low SCFA and NLRP3 activation is still missing. One of the speculations about the bridge is reactive oxygen species (ROS) generated in the state characterized by deficient dietary fiber-derived SCFA. Recent studies demonstrated that sleep deprivation leads to the accumulation of ROS and consequent oxidative stress 49. Another interesting finding revealed the existence of diurnal rhythms in gut flora related to SCFA production 50, 51. Therefore, we speculated that disordered GM-derived SCFA increases ROS, which further contributes to CaMKII phosphorylation and NLRP3 inflammasome activation. However, the potential mechanism explaining the association between SCFA and ROS is yet to be investigated.

Currently, there is a large gap in the area regarding prebiotics and human AF. This study was a preliminary analysis focusing on the dietary fiber-derived SCFA-alleviated AF development and GPR43/CaMKII/NLRP3 signaling. However, further investigation is required to dissect the roles of the SCFA in AF pathogenesis comprehensively. Firstly, the sample size of the cross-sectional study about SCFA quantification was small. Future study with the larger sample size, by continuous feces sample collection from the patient cohort during a follow-up period to acquire the dynamic observation of the dysbiotic GM-related SCFA pattern, might provide strong evidence. Secondly, the potential mechanism underlying a low-fiber diet and activated NLRP3 is yet to be deduced, and experiments related to ROS might provide valuable clues. Thirdly, more experiments disclosing the upstream and downstream mediators of NLRP3 signaling are required to assess the role of SCFA in AF development. Fourthly, further experiments on atrial refractoriness and patch clamp-based ion channel study are needed to assess the impact of SCFA on the electrophysiological characteristics of atrial myocardial cells. Finally, either the harmful effect of a low-fiber diet or the protective role of SCFA on AF pathogenesis cannot be ascribed to a single mechanism, such as low SCFA levels or alleviated NLRP3 inflammasome signaling, respectively. Thus, further studies on non-targeted metabolomic analysis aiming to explore metabolites differentially enriched in low- or high-fiber diet and experiments performed in cardiomyocyte-knockdown of NLRP3 to assess the proportion of inhibited NLRP3 activation with respect to the protective role of SCFA on AF could provide objective and comprehensive acknowledgments.

## Conclusions

In conclusion, SCFA derived from dietary fiber fermentation by gut commensals alleviates AF development via GPR43/NLRP3 signaling. The current findings provide evidence that the activation of atrial GPR43 by SCFA is a promising therapeutic strategy for AF.

## Supplementary Material

Supplementary figures and table.

## Abbreviations

AF: atrial fibrillation
α-SMA: alpha smooth muscle actin
Ca^2+^: calcium
CaMKII: calmodulin-dependent protein kinase II
Cx43: connexin 43
ECG: electrocardiogram
GM: gut microbiota
GC-MS: gas chromatography-mass spectrometry
GPR43: G-protein-coupled receptor 43
IL-1β: interleukin-1 beta
LA: left atrial
LPS: lipopolysaccharides
NLRP3: NACHT, LRR and PYD domain containing protein 3
PAF: paroxysmal AF
psAF: persistent AF
PCoA: principal coordinates analysis
RyR2: ryanodine receptor 2
SCFA: short-chain fatty acid
SEM: standard error of mean
TGF-β: transforming growth factor-beta
