# Supplementary material for: Commensal microbe-derived SCFA alleviates atrial fibrillation via GPR43/NLRP3 signaling
Source: Int J Biol Sci. 2022 Jun 27;18(10):4219–32. doi: 10.7150/ijbs.70644 (PMC9274492; doi:10.7150/ijbs.70644)
Supplement: Supplementary file 1 — Supplementary figures and table. [file ijbsv18p4219s1.pdf]

Table S1

Baseline clinical characteristics of participants in study.

Variable	Control (n=24)	AF (n=24)	*P*-value
Age, years	62.96±10.53	60.08±13.48	0.414
BMI, kg/m^2	26.05 (25.08, 27.82)	24.97 (22.66, 29.41)	0.145
Male	14 (58.33%)	14 (58.33%)	1.000
HTN	19 (79.17%)	13 (54.17%)	0.125
DM	5 (20.83%)	2 (8.33%)	0.416
Smoking	8 (33.33%)	6 (25.00%)	0.752
Drinking	10 (41.67%)	5 (20.83%)	0.212
TG, mmol/L	1.11 (0.91, 1.98)	1.16 (0.87, 1.37)	0.617
TC, mmol/L	4.31±0.89	4.12±0.86	0.475
LDL-C, mmol/L	2.82±0.77	2.72±0.82	0.676
AST, U/L	17.33±5.63	20.75±7.86	0.094
ALT, U/L	14.30 (10.30, 18.70)	17.50 (12.50, 25.50)	0.057
sCr, μmol/L	69.65±13.42	69.57±12.36	0.983

Data are shown as mean ± SD, median (quartile) or number (percentage). ALT, alanine aminotransfease; AST, aspartate aminotransferase; BMI, body mass index; DM, diabetes mellitus; HTN, hypertension; LDL-C, low density lipoprotein cholesterol; sCr, serum creatinine; TC, total cholesterol; TG, triglyceride.

Figure S1

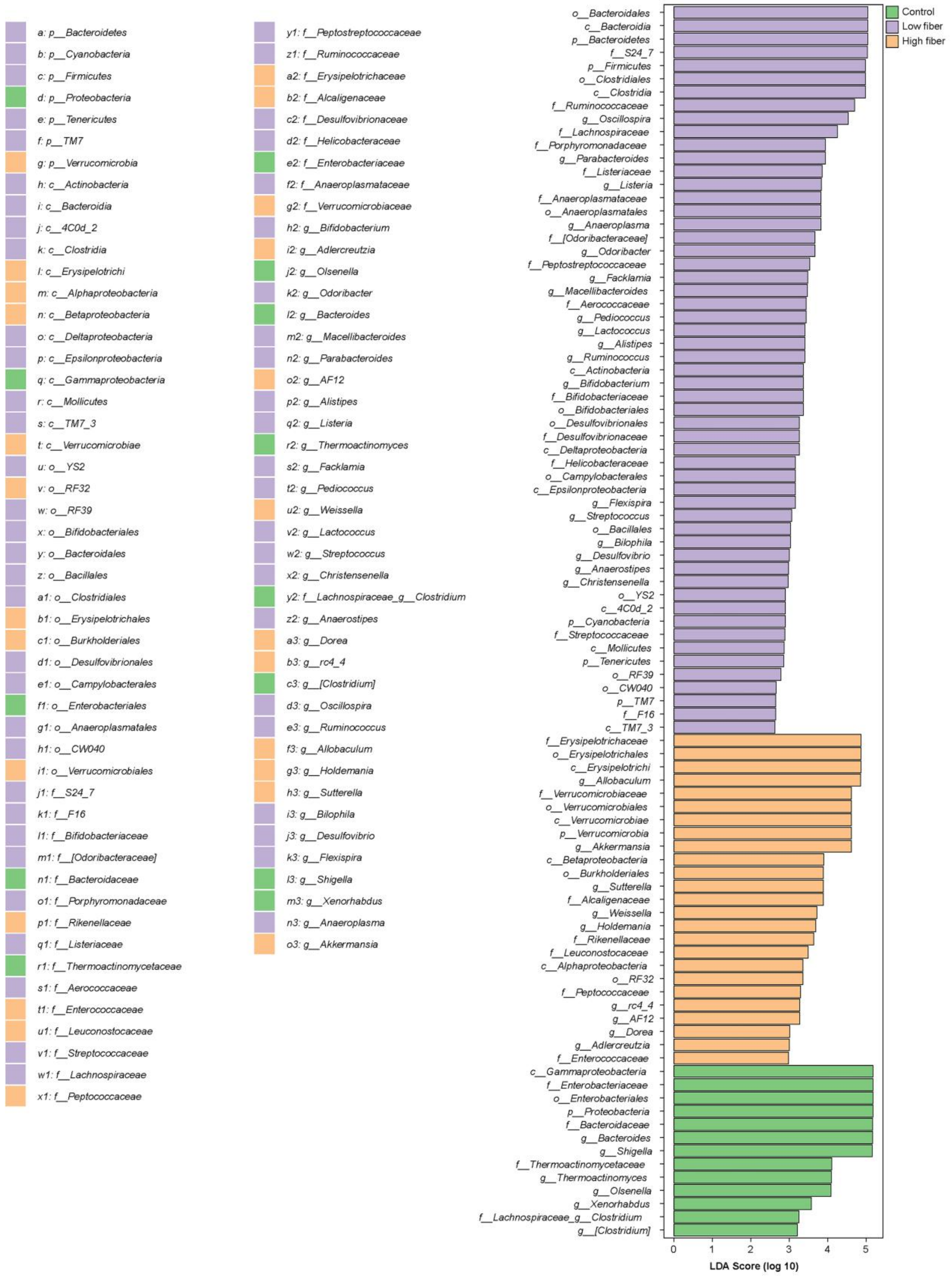
a: p__Bacteroidetes
b: p__Cyanobacteria
c: p__Firmicutes
d: p__Proteobacteria
e: p__Tenericutes
f: p__TM7
g: p__Verrucomicrobia
h: c__Actinobacteria
i: c__Bacteroidia
j: c__4C0d_2
k: c__Clostridia
l: c__Erysipelotrichi
m: c__Alphaproteobacteria
n: c__Betaproteobacteria
o: c__Deltaproteobacteria
p: c__Epsilonproteobacteria
q: c__Gammaproteobacteria
r: c__Mollicutes
s: c__TM7_3
t: c__Verrucomicrobiae
u: o__YS2
v: o__RF32
w: o__RF39
x: o__Bifidobacteriales
y: o__Bacteroidales
z: o__Bacillales
a1: o__Clostridiales
b1: o__Erysipelotrichales
c1: o__Burkholderiales
d1: o__Desulfovibrionales
e1: o__Campylobacterales
f1: o__Enterobacteriales
g1: o__Anaeroplasmatales
h1: o__CW040
i1: o__Verrucomicrobiales
j1: f__S24_7
k1: f__F16
l1: f__Bifidobacteriaceae
m1: f__[Odoribacteraceae]
n1: f__Bacteroidaceae
o1: f__Porphyromonadaceae
p1: f__Rikenellaceae
q1: f__Listeriaceae
r1: f__Thermoactinomycetaceae
s1: f__Aerococcaceae
t1: f__Enterococcaceae
u1: f__Leuconostocaceae
v1: f__Streptococcaceae
w1: f__Lachnospiraceae
x1: f__Peptococcaceae
y1: f__Peptostreptococcaceae
z1: f__Ruminococcaceae
a2: f__Erysipelotrichaceae
b2: f__Alcaligenaceae
c2: f__Desulfovibrionaceae
d2: f__Helicobacteraceae
e2: f__Enterobacteriaceae
f2: f__Anaeroplasmataceae
g2: f__Verrucomicrobiaceae
h2: g__Bifidobacterium
i2: g__Adlercreutzia
j2: g__Olsenella
k2: g__Odoribacter
l2: g__Bacteroides
m2: g__Macellibacteroides
n2: g__Parabacteroides
o2: g__AF12
p2: g__Alistipes
q2: g__Listeria
r2: g__Thermoactinomyces
s2: g__Facklamia
t2: g__Pediococcus
u2: g__Weissella
v2: g__Lactococcus
w2: g__Streptococcus
x2: g__Christensenella
y2: f__Lachnospiraceae_g__Clostridium
z2: g__Anaerostipes
a3: g__Dorea
b3: g__rc4_4
c3: g__[Clostridium]
d3: g__Oscillospira
e3: g__Ruminococcus
f3: g__Allobaculum
g3: g__Holdemania
h3: g__Sutterella
i3: g__Bilophila
j3: g__Desulfovibrio
k3: g__Flexispira
l3: g__Shigella
m3: g__Xenorhabdus
n3: g__Anaeroplasma
o3: g__Akkermansia
o__Bacteroidales
c__Bacteroidia
p__Bacteroidetes
f__S24_7
p__Firmicutes
o__Clostridiales
c__Clostridia
f__Ruminococcaceae
g__Oscillospira
f__Lachnospiraceae
f__Porphyromonadaceae
g__Parabacteroides
f__Listeriaceae
g__Listeria
f__Anaeroplasmataceae
o__Anaeroplasmatales
g__Anaeroplasma
f__[Odoribacteraceae]
g__Odoribacter
f__Peptostreptococcaceae
g__Facklamia
g__Macellibacteroides
f__Aerococcaceae
g__Pediococcus
g__Lactococcus
g__Alistipes
g__Ruminococcus
c__Actinobacteria
g__Bifidobacterium
f__Bifidobacteriaceae
o__Bifidobacteriales
o__Desulfovibrionales
f__Desulfovibrionaceae
c__Deltaproteobacteria
f__Helicobacteraceae
o__Campylobacterales
c__Epsilonproteobacteria
g__Flexispira
g__Streptococcus
o__Bacillales
g__Bilophila
g__Desulfovibrio
g__Anaerostipes
g__Christensenella
o__YS2
c__4C0d_2
p__Cyanobacteria
f__Streptococcaceae
c__Mollicutes
p__Tenericutes
o__RF39
o__CW040
p__TM7
f__F16
c__TM7_3
f__Erysipelotrichaceae
o__Erysipelotrichales
c__Erysipelotrichi
g__Allobaculum
f__Verrucomicrobiaceae
o__Verrucomicrobiales
c__Verrucomicrobiae
p__Verrucomicrobia
g__Akkermansia
c__Betaproteobacteria
o__Burkholderiales
g__Sutterella
f__Alcaligenaceae
g__Weissella
g__Holdemania
f__Rikenellaceae
f__Leuconostocaceae
c__Alphaproteobacteria
o__RF32
f__Peptococcaceae
g__rc4_4
g__AF12
g__Dorea
g__Adlercreutzia
f__Enterococcaceae
c__Gammaproteobacteria
f__Enterobacteriaceae
o__Enterobacteriales
p__Proteobacteria
f__Bacteroidaceae
g__Bacteroides
g__Shigella
f__Thermoactinomycetaceae
g__Thermoactinomyces
g__Olsenella
g__Xenorhabdus
f__Lachnospiraceae_g__Clostridium
g__[Clostridium]
0
1
2
3
4
5
LDA Score (log 10)
Control
Low fiber
High fiber

Figure S2

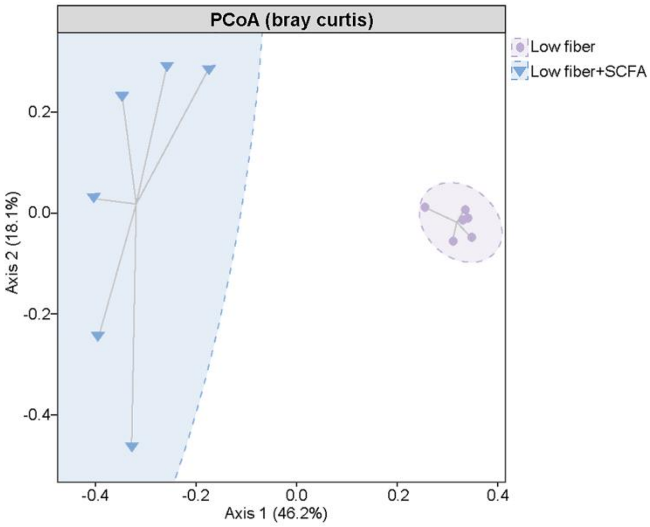
PCoA (bray curtis)
Low fiber
Low fiber+SCFA
0.2
0.0
-0.2
-0.4
Axis 2 (18.1%)
-0.4
-0.2
0.0
0.2
0.4
Axis 1 (46.2%)

Figure S3

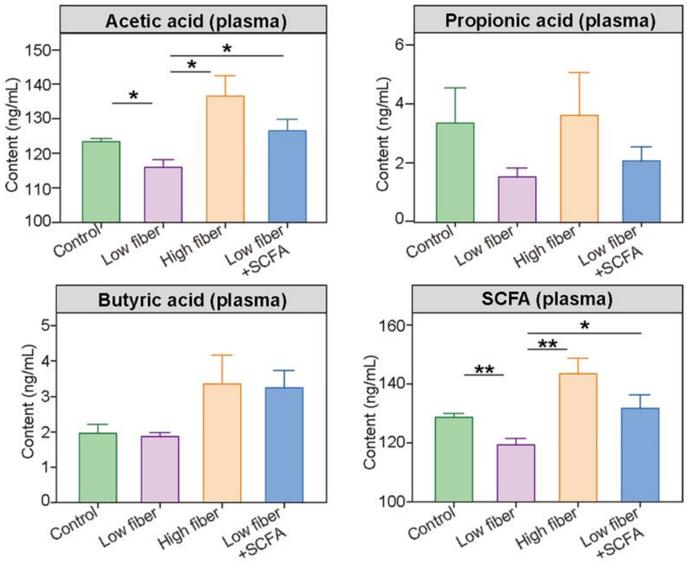
Acetic acid (plasma)
Content (ng/mL)
150
140
130
120
110
100
*
*
*
Control
Low fiber
High fiber
Low fiber +SCFA
Propionic acid (plasma)
Content (ng/mL)
6
4
2
0
Control
Low fiber
High fiber
Low fiber +SCFA
Butyric acid (plasma)
Content (ng/mL)
5
4
3
2
1
0
Control
Low fiber
High fiber
Low fiber +SCFA
SCFA (plasma)
Content (ng/mL)
160
140
120
100
**
**
*
Control
Low fiber
High fiber
Low fiber +SCFA

Figure S4

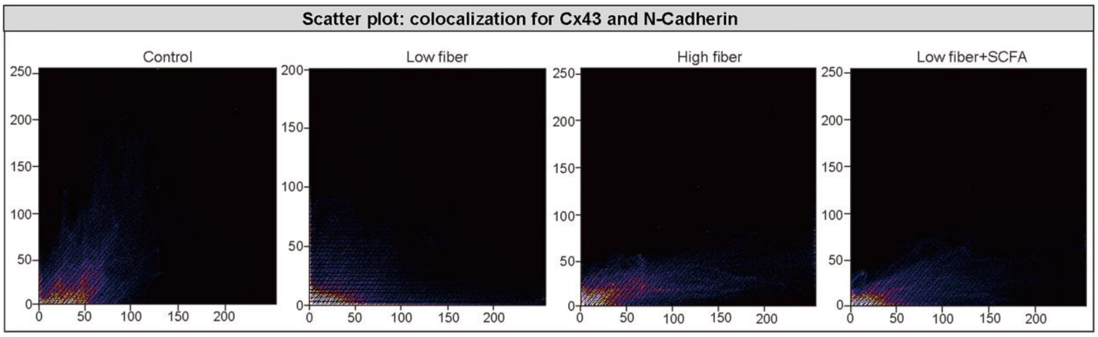
Scatter plot: colocalization for Cx43 and N-Cadherin
Control
Low fiber
High fiber
Low fiber+SCFA
0
50
100
150
200
250

Figure S5

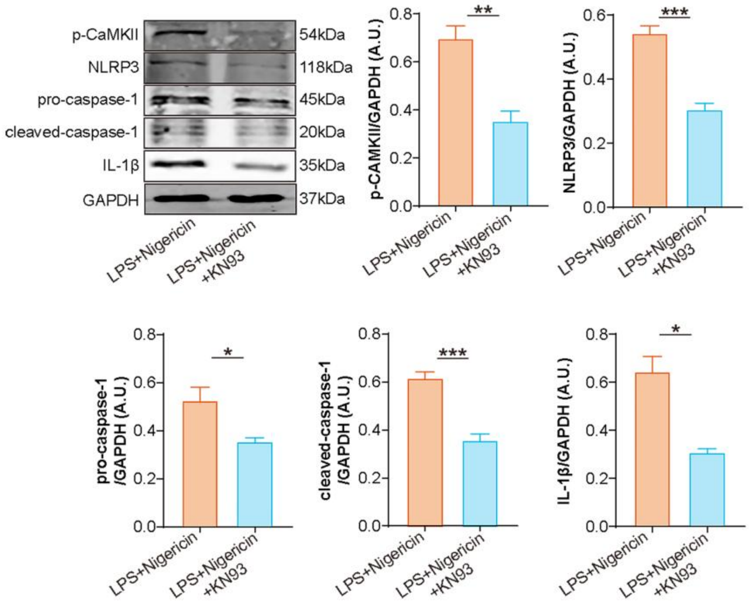

p-CaMKII
NLRP3
pro-caspase-1
cleaved-caspase-1
IL-1β
GAPDH
54kDa
118kDa
45kDa
20kDa
35kDa
37kDa
LPS+Nigericin
LPS+Nigericin +KN93
p-CAMKII/GAPDH (A.U.)
NLRP3/GAPDH (A.U.)
pro-caspase-1 /GAPDH (A.U.)
cleaved-caspase-1 /GAPDH (A.U.)
IL-1β/GAPDH (A.U.)
**
***
*
***
*
